# Pixel-level plasmonic microcavity infrared photodetector

**DOI:** 10.1038/srep25849

**Published:** 2016-05-16

**Authors:** You Liang Jing, Zhi Feng Li, Qian Li, Xiao Shuang Chen, Ping Ping Chen, Han Wang, Meng Yao Li, Ning Li, Wei Lu

**Affiliations:** 1 National Laboratory for Infrared Physics, Shanghai Institute of Technical Physics, Chinese Academy of Sciences, 500 Yutian Road, Shanghai 200083, P. R. China; 2 Synergetic Innovation Center of Quantum Information & Quantum Physics, University of Science and Technology of China, Hefei, Anhui 230026, P. R. China

## Abstract

Recently, plasmonics has been central to the manipulation of photons on the subwavelength scale, and superior infrared imagers have opened novel applications in many fields. Here, we demonstrate the first pixel-level plasmonic microcavity infrared photodetector with a single quantum well integrated between metal patches and a reflection layer. Greater than one order of magnitude enhancement of the peak responsivity has been observed. The significant improvement originates from the highly confined optical mode in the cavity, leading to a strong coupling between photons and the quantum well, resulting in the enhanced photo-electric conversion process. Such strong coupling from the localized surface plasmon mode inside the cavity is independent of incident angles, offering a unique solution to high-performance focal plane array devices. This demonstration paves the way for important infrared optoelectronic devices for sensing and imaging.

There is growing demand for high-performance infrared imagers for many applications, such as remote sensing^1^, non-invasive medical diagnostics^2^, astronomy^3^ and defense^4^. Infrared imagers are developing from first-generation scanning systems of linear arrays and second-generation staring systems of two-dimensional arrays toward third-generation systems of larger numbers of pixels, higher frame rates, better thermal resolution, multicolor and/or other on-chip signal-processing functions^5^. Quantum well infrared photodetector focal plane arrays (QWIP-FPAs) have the potential to be third-generation, high-performance infrared photodetector FPAs due to their high uniformity and operability, larger format and dual band capability, especially in long- and very long-wavelength regions. However, QWIPs are based on intersubband transitions with a low density of states of both the ground state and excited state than that of narrow-bandgap infrared detecting materials (such as HgCdTe) and suffer from weak light absorption and low photo-electric conversion efficiency^6^. A series of optical coupling structures has been developed to improve the light absorption efficiency^7^,^8^. Surface plasmons (SPs) have attracted increasing interest for enhancing the performance of quantum wells/quantum dots infrared photodetectors due to the artificially manipulated propagation and concentration of photons^9^,^10^. Enhanced photo-responsivity has been reported^11^,^12^,^13^. When applied to detecting pixels of FPA at very long-wavelength range (12–30 μm), however, because of the pixel sizes of 20–30 μm, SP coupling may suffer from low enhancement due to too few periods within each pixel as the periodicity that supports the surface plasmons is broken by the boundaries of pixels, yielding low SPP coupling. SP coupling is also sensitive to incident angle owing to its incident angle-related wavevector matching mechanism.

Alternatively, plasmonic microcavities have also been the basis of manipulating photons in the subwavelength scale for various applications, such as lasers^14^,^15^, explosives detection^16^ and surface-enhanced Raman scattering^17^,^18^. In its application in photodetection, although plasmonic microcavity infrared photo-detectors have been reported^19^, the results of the responsivity enhancement have not been given. The photosensitive area is 200 × 200 μm^2^, rather than the common pixel size of FPA. Whereas the large area plasmonic microcavity has very good photon capture and confinement capabilities where the photo-environment can be regarded as infinite, for small pixels with sizes of only 2–3 times that of the free space wavelength where the diffraction or scattering from pixel boundaries may occur and damage these capabilities, questions remain regarding whether their characteristics will be preserved. Here, we report the first pixel-level plasmonic microcavity quantum well infrared photodetectors (PMC-QWIPs) based on a metal/dielectric/metal (MIM) structure by sandwiching a single quantum well between the metal patches and the metal reflection layer, with a photosensitive area of 27 × 27 μm^2^, the same as pixels in FPA, as illustrated in Fig. 1. The subwavelength distance between the two metal layers leads to a strong MIM coupling, resulting in optical field localization under the metal patches, forming a cavity localized surface plasmon (LSP) mode resonance. Incident photons are captured and travel laterally back and forth within the double-metal area due to the impendence mismatch at the terminations and are constantly absorbed by the quantum well. The capture and compression of photons greatly increase the number of photons in the plasmonic microcavity, decreasing the escape rate, resulting in over one order of magnitude enhancement of the peak responsivity of the PMC-QWIPs. We demonstrate, by measuring the photocurrent spectra of the PMC-QWIP under different incident angles, that the cavity LSP mode resonant wavelength is fixed at the same wavelength due to the characteristics of the cavity mode. Moreover, by using *X-Y* direction symmetric metal patches, the PMC-QWIP is insensitive to the incident polarization, which indicates that after going through the infrared focusing system, as much as photons with different incident angles and polarizations can be captured by the plasmonic microcavity, creating enhanced, stable and uniform optical signals and greatly improving the FPA device. A series of one-dimensional PMC-QWIP with different pixel areas from 200 × 200 μm^2^ to 20 × 20 μm^2^ is designed to clarify the pixel size effect on optical coupling capability of the cavity LSP mode and of the surface plasmon polariton (SPP) mode. As the pixel size is reduced close to the detection wavelength (approximately 13 μm), the SPP mode in larger area pixels becomes relatively weaker, and the cavity LSP mode is still strong. The SPP and cavity LSP mode are determined by the grating periodicity and metal width, respectively. Our results demonstrate that the plasmonic microcavity can surpass the limit of periodicity, in which a large enough photosensitive area is required. In addition, such a plasmonic microcavity is completely compatible with the conventional manufacturing processes of the FPAs. Compared with the minimum lithography size (approximately *λ*_0_/2*n*, where *λ*_0_ is the wavelength in vacuum and *n* is the refractive index of the dielectric) required in conventional dielectric grating or SPs scheme based on metal hole arrays, where the first-order diffraction is employed and higher orders are very weak, the strong enough third-order of the cavity LSP mode used in our plasmonic microcavity implies a larger lithography size (approximately *mλ*_0_/2*n*, where *m* is the order of the resonance and the phase of reflection at the structure termination is neglected), which can be fabricated with inexpensive standard lithography processes, removing the dependence on expensive and inefficient nano-processing procedures.

## Results

### Design and fabrication of the PMC-QWIP

**Figure 1:**
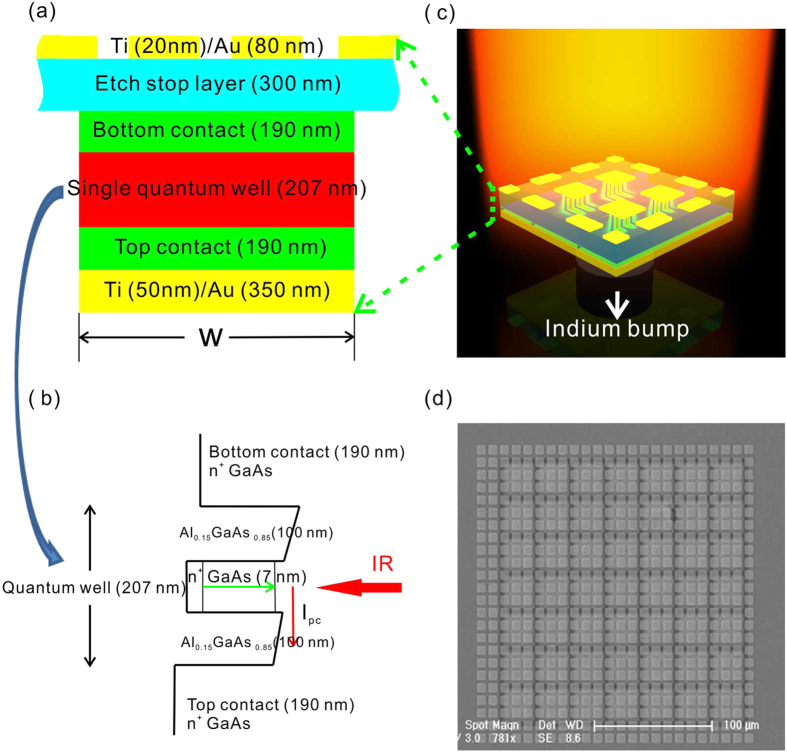
Schematic view of the pixel-level PMC-QWIP. (**a**) Cross-sectional view of a single pixel. (**b**) Band diagram of the quantum well and the thicknesses of the sub-layers. (**c**) PMC-QWIP hybridized with a fanout circuit. (**d**) SEM image of the PMC-QWIP.

Figure 1 is a schematic view of the MIM PMC-QWIP. Figure 1a shows the cross-sectional diagram of the PMC-QWIP pixel. A single quantum well is sandwiched by square metal patches and a metal reflection layer. Figure 1b shows the conduction band diagram of the quantum well and the thicknesses of the sub-layers. Heavy doped top/bottom contact layers on both sides of the quantum well are required to realize photo-electric detection. The dielectric layer thickness between the metal patches and the reflection layer is fixed at 887 nm to form a plasmonic microcavity. The photo-detecting pixel of the PMC-QWIP is shown in Fig. 1c, and approximately 2 × 2 metal patches in each single pixel can form plasmonic microcavities with the metal reflection layer. The fabricated pixel array is hybridized with a fanout circuit through a standard flip-chip bonding process. The size of the pixel is fixed at 27 × 27 μm^2^, and the period of the pixels in the 2D array is 30 μm, where there are approximately 3-μm separations between adjacent pixels. Figure 1d shows the top view of the SEM image after fanout circuit bonding, substrate removal and metal patch deposition. The detailed processing sequence can be found in the Supplementary Information (SI).

### Experimental and simulated plasmonic microcavity resonance mode

**Figure 2:**
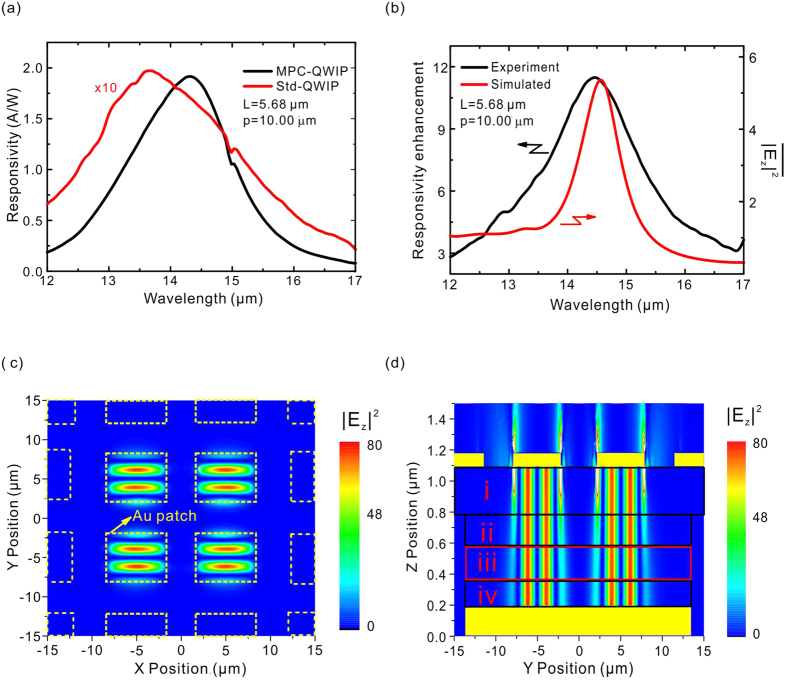
Responsivity and LSP mode field maps of the PMC-QWIP. (**a**) Responsivity spectra of the PMC-QWIP (black line) and standard device (red line). The spectra were acquired at the operating temperature of 35 K and the bias voltage of 0.3 V. (**b**) Responsivity relative intensity spectrum of the PMC-QWIP (black line) and the simulated 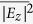 in the QW active layer (red line). (**c**) Simulated distribution of 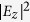
in the *XOY* plane (*z* = 0.5 μm) of LSP mode. (**d**) Simulated distribution of 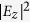 in the *YOZ* plane (*x* = 5 μm), where areas i, ii, iii and iv correspond to the etch stop layer, bottom contact, quantum well active layer and top contact, respectively.

The photo-responsivity spectra of the PMC-QWIP are obtained from Fourier transform infrared photocurrent spectra together with the blackbody responsivity. The experimental setup and data calculation can be found in the SI. Figure 2a shows a typical spectrum as a black line. Also shown is the responsivity spectrum (×10) of the 45 degree edge facet coupled device^6^ (red line) from the same wafer. The spectra are acquired at the operating temperature of 35 K and bias voltage of 0.3 V. Because the latter is not modulated by any artificial structures, it reflects the intrinsic responses of the quantum well intersubband transition and is commonly regarded as the standard device. Under the modulation of the plasmonic microcavity, the responsivity peak extends from 13.7 μm to 14.3 μm and approximately one order of magnitude enhancement is observed. This strong enhancement effect of the photo-electric response is attributed to the incident photon manipulations of the plasmonic microcavity. Incident photons are coupled into the microcavity at the metal patch edges and are modulated by the cavity mode. The propagation direction is changed from the *z*-direction in free space to the *x-y* direction in the cavity. The distribution of the optical field is also modulated by the microcavity to form a strong confined optical field. Due to the quantum transition rule, photons with electric field components along the *z*-direction are required for being absorbed by electrons in the quantum well via intersubband transitions to generate photo-induced carriers. The utilization ratio of photons is greatly improved due to the strong confinement and concentration of optical energies, resulting in an enhanced responsivity of the PMC-QWIP.

To clearly understand the manipulation behavior of the plasmonic microcavity, the responsivity spectrum of the PMC-QWIP has been divided by that of the standard QWIP, yielding the relative enhancement spectrum, in which the intrinsic absorption effect of the quantum well has been eliminated, i.e., the intensity spectrum against wavelength is only determined by the microcavity structures, reflecting the optical modes inside the microcavity. The black line in Fig. 2b shows a divided curve, which forms a single peak. According to our previous theoretical and experimental results^20^, this peak is attributed to the cavity LSP mode. The peak is at the wavelength of 14.5 μm, close to the peak wavelength of the responsivity of the PMC-QWIP (14.3 μm). The value of the peak intensity reflects the enhancement factor of the QWIP responsivity promoted by the plasmonic microcavity.

To theoretically verify the attribution of the cavity LSP mode, the COMSOL Multiphysics simulation platform is employed to numerically calculate the optical energy distributions. The complete detecting pixel is set as the simulation area with the perfect match layer boundaries in three directions (the selection of the boundary condition is described in the SI), and the normal incident electric field is taken as 1. According to the quantum transition rule, only the photons with an electric *z*-component can be absorbed by the quantum well. The experimentally measured responsivity enhancement spectrum of the PMC-QWIP represents the average of the square of the electric field along the *z*-direction within the quantum well active layer, which can be expressed as follows:


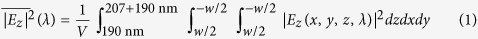


where *V* is the QW absorption volume, *V* = 0.207 × 27 × 27 μm^3^ and *w* = 27 μm is the pixel width (as indicated in Fig. 1a). The simulated results are plotted in Fig. 2b as a red line. Within the wavelength range from 12–18 μm, a single peak at 14.5 μm is observed, in good agreement with the experimental peak of the cavity LSP mode. The simulation reproduces the mode spectrum of photons inside the microcavity and confirms that the experimental responsivity enhancement is originated from the cavity LSP mode.

To further reveal the mode properties in the microcavity, the distribution of optical energy for *y*-polarized light at the peak wavelength of 14.5 μm of the cavity LSP mode is simulated using the same theoretical methods. Figure 2c,d describe the distribution of the square of the electric field along the *z*-direction in the *XOY* plane (*z* = 0.5 μm) and *YOZ* plane (*x* = 5μm), respectively. Most of optical energy is confined in the plasmonic microcavity formed by the central metal patches and the metal reflection layer. The peripheral patches on the pixel are not completely contained within the reflection layer; therefore, the resonance conditions are not satisfied for creating the cavity LSP mode and the electric field underneath is weak. For the central cavities, the optical fields are significantly enhanced. The maximum value of the square of the electric field along the *z*-direction can be over 80 in the quantum well active region, as indicated by the red box (region iii) in Fig. 2d. The optical energy along the *z*-direction between the two metal layers is uniform, especially within the region iii, benefiting from the MIM enhancement, which provides convenience for the design of the material structure, in contrast to the single perforated metal layer SPs schemes that require the active layer to be close to the near-field region. The simulated optical energy distribution in Fig. 2c,d demonstrates that the incident photons are captured and confined by the plasmonic microcavities, forming the strong optical field localization, leading to an improved photon utilization ratio in the photo-electric conversion. However, although the strongest *E*_*z*_ field appears at the edges of the central patches due to the in-plane enhancement, the energy is not converted into photocurrent because there is no overlap with the QW. It is a challenging task to transfer edge-energy to the active region in the future optimizations.

The Fabry-Perot (F-P) resonance model^19^,^20^ is used to explain the resonance mechanism in such a metal-patch or metal-strip plasmonic microcavity. At the edges of metal patches, the incident *y*-polarized light is coupled and transformed into the MIM plasmonic waveguide mode propagating along the *y*-direction. Due to the impendence mismatch at the boundaries between single/double metal regions, reflection occurs when the waveguide mode meets the lateral interfaces, and the modulus and phase of the reflection coefficient are defined by the structure and materials. A standing wave is formed when the optical path of the waveguide mode traveling along the *y*-direction per round is a multiple of *λ*_0_/*n*_eff_ (*n*_eff_ is the effective refractive index of the MIM waveguide, *λ*_0_ is the wavelength in vacuum), leading to an increased lifetime of photons staying in the active layer and compressed optical energy. COMSOL simulation has fully described the propagation and reflection of the waveguide mode; the details can be found in SI Figure S4. Because the quantum well exhibits only weak absorption, and the simulated propagation length can exceed 50 μm (as shown in SI Figure S4d), photons can travel back and forth over a long enough distance to support confined optical field. Supplementary Movie 1 shows a live process in which photons are captured in the plasmonic microcavities from free space to form a strong cavity LSP mode. Supplementary Movie 2 shows a comparison of imaging between the PMC-QWIP and the Std-QWIP with a moving cross hole screen placed in front of a blackbody light source. The imaging quality is greatly improved by the PMC-QWIP.

### Cavity LSP mode, incident angle and polarization characteristics of PMC-QWIP

**Figure 3:**
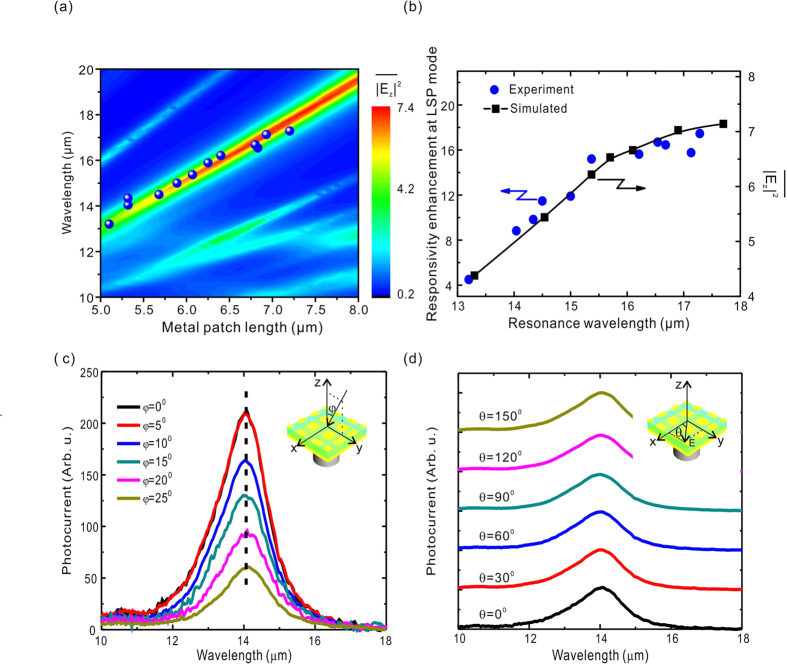
Cavity LSP mode, incident angle and polarization characteristics of the PMC-QWIP. (**a**) Experimental resonance wavelength dependence on the metal patch. Also shown is the simulated map of 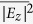 in the QW layer as a function of the metal patch length *L* and wavelength. (**b**) Experimental responsivity enhancement factor (blue dots) of the cavity LSP mode (taken from the responsivity peaks of the PMC-QWIP devices with different metal patches) and the simulated 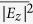 of the LSP mode in the QW layer (black square) as a function of the resonance wavelength. The line is to guide the eye. (**c**) Photocurrent spectra under different incident angles. (**d**) Photocurrent spectra with different incident polarizations. Curves are vertically shifted for clarity.

To verify the F-P model in experiment and to further reveal the photon manipulation of the microcavity, a series of PMC-QWIP with different metal patch lengths are fabricated. Their responsivity spectra are measured, and the dominance of the metal patch size on the resonance wavelength and the peak responsivity is observed. Figure 3a plots the dependence of the resonance wavelength on the metal patch lengths; the solid blue circles are taken from the experimental peak wavelengths of the cavity LSP mode (as that shown in Fig. 2b). The resonance wavelength is redshifted almost linearly as the metal patch gets larger, indicating the patch length regulation. The background colored graph shows the map of 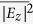 in the QW layer as a function of the metal patch length *L* and wavelength. The maxima of 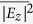 matches the experimental resonance wavelengths at different patch lengths. The optical energy increases as the resonance wavelength gets longer, in good agreement with the simulated results in SI Figure S4, in which the reflectance at the terminations of the structure increases at longer wavelength, resulting in tighter confinement of photons inside the cavity and stronger optical field squeezing. Figure 3b presents the dependence of the responsivity enhancement factor on the resonance wavelength. The values of the enhancement factors in solid blue dots are taken from the experimental peaks, as the one shown in Fig. 2b, whereas the solid black squares are the simulated values of 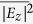 of the cavity LSP mode in the QW layer. The solid line is to guide sight. The responsivity enhancement factor increases almost linearly from approximately 4.5–15 as the resonance wavelength red-shifts from 13.2–15.7 μm. However, the value tends to saturate after 15.7 μm. The simulation agrees with the experiments.

We have analyzed the resonance conditions to explain the increase and saturation of the enhancement factor. In principle, optical coupling capability can be optimized by tuning the geometric parameters (such as the dielectric layer thickness and the metal patch length) to reach a resonant point for a specific wavelength. Limited by the photodetector structure (containing top- and bottom-contact, the quantum well active layer and the etch stop layer) together with the processing precision; however, the thickness of the total dielectric is difficult to adjust to match various geometric resonance conditions. In the experiment, the thickness is usually fixed, and the optimum resonance condition corresponds to one specific wavelength. For other wavelengths, there are deviations of 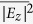 between the experimental and theoretical geometric parameters. This deviation dependence on the metal patch length has been calculated, and the details can be found in SI Figures S5 and S6. The simulated results show that as the wavelength gets longer, the experimental geometric parameters are closer to the resonance, and the deviation approaches zero, as shown by the solid squares in Fig. 3b. Within the experimental patch size range, the enhancement factor saturates at wavelengths larger than 15.7 μm. The simulated wavelength dependence of the deviation agrees with the experiments, verifying that the increased enhancement factor at longer wavelength originates from the approach of the geometric parameters to the resonant parameters.

In terms of FPA, the effects of both the incident angle and polarization on the detected signals play important roles in the overall performance of devices. By conducting the angle- and polarization-dependent photocurrent measurements, the response characteristics of the PMC-QWIP are explored. The inset of Fig. 3c illustrates the experimental configuration. The detector normal is along the *z*-axis, whereas the incident angle is *φ*. The curves in Fig. 3c display photocurrents under various incident angles. Although the strength changes, the response peak is fixed at 14.06 μm, showing the independence of the incident angle and indicating only one fixed cavity LSP mode. In FPA devices, the image of the target is focused on detecting pixels with various incident angles and polarizations. Taking the focusing system of F = 2, for example, the FPA is illuminated with incident angles from 0^0^–45^0^. The fixed response peak signifies that the pixels at different positions on the FPA share the same responsive peak to guarantee the wavelength uniformity of the imaging, which is critical in some applications for maintaining the spectrum resolution. Such a fixed peak wavelength in our PMC-QWIP results from the F-P resonance of the MIM cavity LSP mode, in which the extra plane wavevector (strongly dependent on the incident angle) is not required. On the other hand, Fig. 3d presents the photocurrents measured with different polarizations, where *θ* is the polarization angle against the *x*-axis created by a polarizer in front of the detector. The curves are vertically shifted for clarity. The intensities and peaks are not changed with the incident polarization, indicating the insensitivity to polarization, which results from the *X-Y* symmetry of the metal patches.

### Experimental demonstration of cavity LSP mode’s advantage in pixel-level photo-electric devices compared with SPP mode

**Figure 4:**
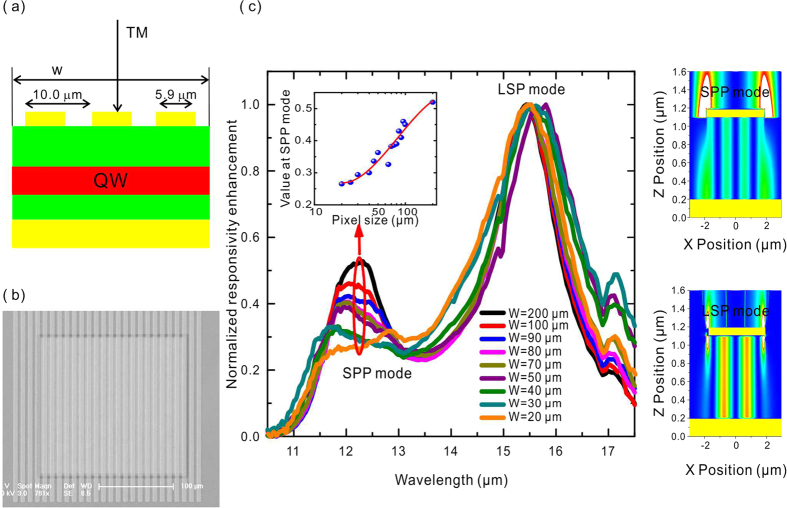
Pixel size dependence of the cavity LSP and SPP mode of the PMC-QWIP. (**a**) Cross-sectional view of the PMC-QWIP illuminated by TM-polarized light. (**b**) SEM image of the PMC-QWIP with a pixel size of *w* = 200 μm. The period and strip width are fixed at 10 and 5.9 μm, respectively. (**c**) Normalized responsivity enhancement spectra with different pixel size *w*. The inset shows the SPP mode peak values as a function of *w*. The right-hand panel is for the distributions of 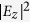 of the SPP and LSP modes.

Compared with our pixel-level PMC-QWIP that offers single cavity LSP mode, our previously reported large area ones exhibit the co-existence of LSP and SPP mode^20^. This co-existence implies a pixel size effect on the optical modes. To determine the details and the effects on the optical coupling capabilities, a series of one-dimensional PMC-QWIPs with different square pixel sizes from 200×200 μm^2^ to 20 × 20 μm^2^ are fabricated, and they share the same 1D metal grid grating with a period of 10.0 μm and a strip width of 5.9 μm. Figure 4a,b are the schematic cross-sectional diagram and top SEM image, respectively. Figure 4c describes the normalized responsivity spectra. Two resonant peaks at 12.3 μm and 15.5 μm are observed, and the corresponding distributions of the electric field are shown in the right panel insets in Fig. 4c, which can be attributed to SPP and cavity LSP mode resonances, respectively. Their intensities are normalized to the peak of the cavity LSP mode. As the pixel size approaches the detecting wavelength, the peak intensity of the SPP mode becomes weaker. The inset curve shows the peak height of the SPP mode against the pixel size. The size effect of the cavity LSP and SPP mode might originate from their different activation mechanisms. The SPP mode can be excited by a periodic grating vector. With small pixel size, the grating periodicity is broken, and the plane wavevector diminishes, leading to weak SPP coupling. However, the cavity LSP mode can be excited in single plasmonic microcavity, remaining strong even when the pixel size is close to the detecting wavelength. Therefore, the plasmonic microcavity with the MIM cavity LSP coupling is especially beneficial for long- and very long-wavelengths, even THz FPA, where the single layered SPs coupling scheme may fail.

## Discussion

We have reported the first pixel-level plasmonic microcavity infrared photodetectors by integrating the MIM structure into the QWIP pixel. Greater than one order of magnitude enhancement of the peak responsivity has been observed. The simulated optical field proves that the enhancement originates from strong optical energy confinement in the microcavity between the metal patch and the metal reflection layers, forming strong cavity LSP-QWIP coupling. The semi-analytical Fabry-Perot model reveals the optical field distribution and the determination of the resonance wavelength in the microcavity, providing an analytical equation for estimating the resonance wavelength. Moreover, the PMC-QWIP pixel can offer a fixed optical microcavity mode insensitive to the incident angle and polarization, breaking the limit of periodicity required in the SPs scheme, which is beneficial to the response uniformity of FPA devices. Study of the effect of the pixel size on the optical coupling capability of cavity LSP and SPP mode shows that as the pixel width decreases and approaches the detecting wavelength, the SPP mode becomes relatively weaker, whereas the cavity LSP mode remains strong. Although the total quantum efficiency (QE) of the PMC-QWIP device is low (approximately 8%) due to the use of single QW, it may be further improved by introducing more QWs and/or reducing the dark current. Because the QE is determined by both photo-absorption and carrier transport, the current MIM structure has been proved to provide significant enhancement of photo-absorption, which improves QE at the optical side. This work demonstrates that the plasmonic microcavity offers FPA as a better optical solution to generate the enhanced, uniform, and stable photo-electric signals from three aspects of peak responsivity enhancement, incident angle and polarization independences. Whereas the resonance wavelength can be easily modulated by tailoring the microcavity length, future works should be focused on realizing the selection of the detection wavelength by changing the geometric parameters of the plasmonic microcavity while the electronic states of the detecting material are unchanged. In other words, pixel-level spectral resolution can be achieved by integrating metal patches with different patch lengths into different pixels. Therefore, this method holds promise for miniaturized inexpensive and easy-to-use on-chip spectrometers in applications sensitive to size, weight, cost and complexity. With the convenience of replacing metal patches with strips, the FPAs have polarization sensing capability and can be used as powerful multimodal spectropolarimetric sensors.

## Methods

### Fabrication of pixel-level plasmonic microcavity infrared photodetectors

The fabrication of pixel-level PMC-QWIPs was initiated on GaAs wafers. An 887 nm-thick dielectric layer was grown on a GaAs wafer by molecular beam epitaxy (MBE). According to the order of growth, the dielectric layer contained the following: a 300 nm-thick n-doped Al_0.4_Ga_0.6_As etch stop layer (n = 2 × 10^17^ cm^−3^), a 190 nm-thick n-doped GaAs bottom contact layer (n = 2 × 10^17^ cm^−3^), a 207 nm-thick single quantum layer (100 nm Al_0.15_Ga_0.85_As/7 nm n-doped GaAs/ 100 nm Al_0.15_Ga_0.85_As) and a 190 nm-thick n-doped GaAs top contact layer (n = 2 × 10^17^ cm^−3^). The common electrode was prepared by wet chemical etching, and the mesa depth was controlled to reach the bottom contact layer. A GeAu (100 nm)/Ni (20 nm)/Au (400 nm) layer was deposited by electron-beam evaporation, and the top and bottom electrodes were formed by a lift-off process. Then, rapid thermal annealing was performed to form Ohmic contact. A Ti (50 nm)/Au (300 nm) was deposited by electron-beam evaporation as the metal reflection layer of the MIM structure. A 300 nm-thick SiN_*x*_ passivation layer was deposited by plasma-enhanced chemical vapor deposition to provide physical and chemical protection. The fabricated chip was hybridized with a fanout circuit through a standard flip-chip bonding process. An underfill epoxy process was provided to protect the device. The chip was thinned to 20–30 μm by a mechanical polishing process, and a high-selectivity recipe (mixture of citric acid (C_6_H_8_O_7_) and hydrogen peroxide (H_2_O_2_)) was adopted to remove the rest of the GaAs from the etch stop layer. Finally, a metal patch array was formed by electron-beam evaporation and lift-off process, and the pixel-level PMC-QWIP device was completed. The flowchart for preparing the PMC-QWIP is shown in SI Figure S1.

## Additional Information

**How to cite this article**: Jing, Y. L. *et al.* Pixel-level plasmonic microcavity infrared photodetector. *Sci. Rep.* **6**, 25849; doi: 10.1038/srep25849 (2016).

## Supplementary Material

Supplementary Information

Supplementary Movie 1

Supplementary Movie 2
