# Supplementary material for: Pixel-level plasmonic microcavity infrared photodetector
Source: Sci Rep. 2016 May 16;6:25849. doi: 10.1038/srep25849 (PMC4867604; doi:10.1038/srep25849)
Supplement: Supplementary Information [file srep25849-s1.pdf]

Supplementary Information

Pixel-level plasmonic microcavity quantum well infrared photodetector

YouLiang Jing, ZhiFeng Li*, Qian Li, XiaoShuang Chen, PingPing Chen, Han Wang, MengYao Li, Ning Li and Wei Lu*

National Laboratory for Infrared Physics, Shanghai Institute of Technical Physics, Chinese Academy of Sciences, 500 Yutian Road, Shanghai 200083, P. R. China

Synergetic Innovation Center of Quantum Information & Quantum Physics, University of Science and Technology of China, Hefei, Anhui 230026, P. R. China

*E-mail: luwei@mail.sitp.ac.cn

*E-mail: zfli@mail.sitp.ac.cn

1. Fabrication

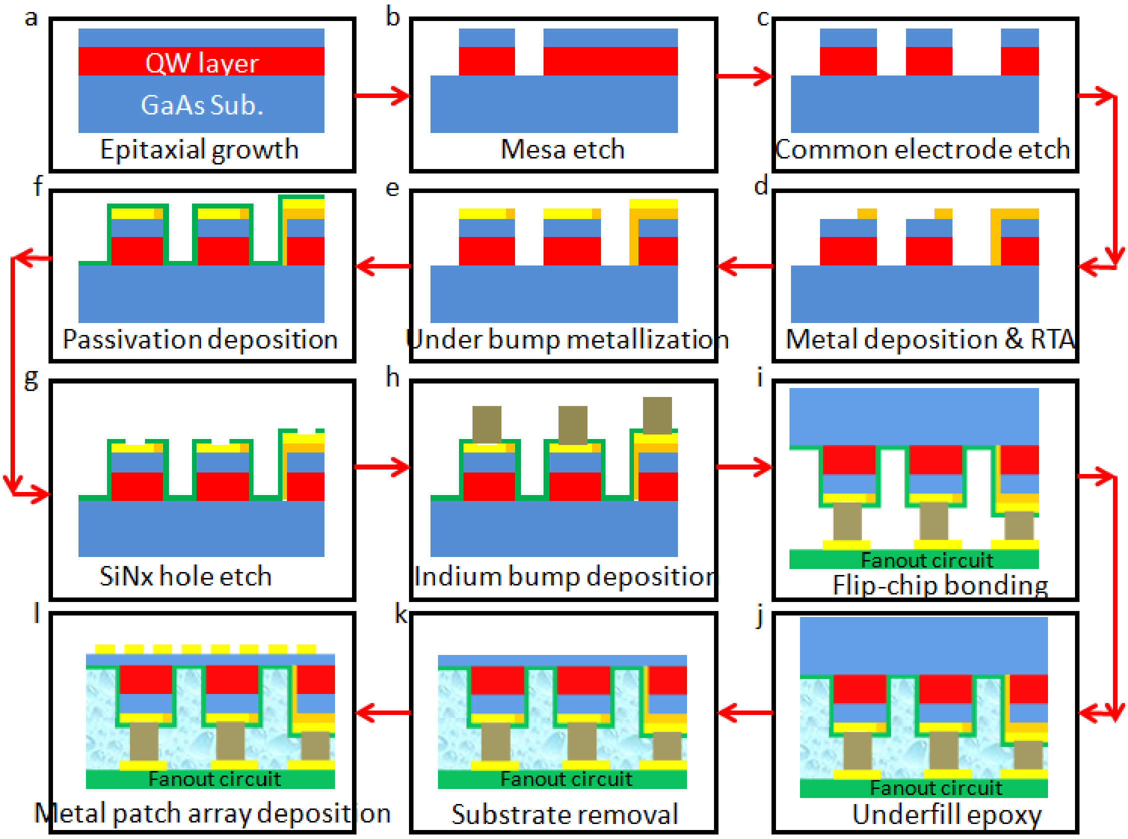

Figure S1 | Schematic presentation of the fabrication procedure of the pixel-level plasmonic microcavity quantum well infrared photodetector

2. Measuring the responsivity spectrum

The responsivity spectrum is obtained by measuring the blackbody responsivity and the photocurrent spectrum of the PMC-QWIP device. The blackbody responsivity is measured with the experimental configuration shown in Fig. S2a. The operating temperature, bias, and blackbody temperature are 35 K, 0.3 V, and 1000 K, respectively. The photocurrent spectrum is measured with Fourier transform infrared spectrometer. The experimental configuration is shown in Fig. S2b, the operating temperature and bias temperature are 35 K and 0.3 V. Thus, we can obtain the responsivity spectrum $R(\lambda)$ by $R(\lambda)=R\times PC(\lambda)$[1], where R is the blackbody responsivity

and PC(λ) is the photocurrent spectrum with its peak value normalized to 1. For the experiment of measuring the photocurrent spectrum of the PMC-QWIP at the oblique incident angle φ, we change the incident angle φ by rotating the cryostat, as shown in Fig. S2b.

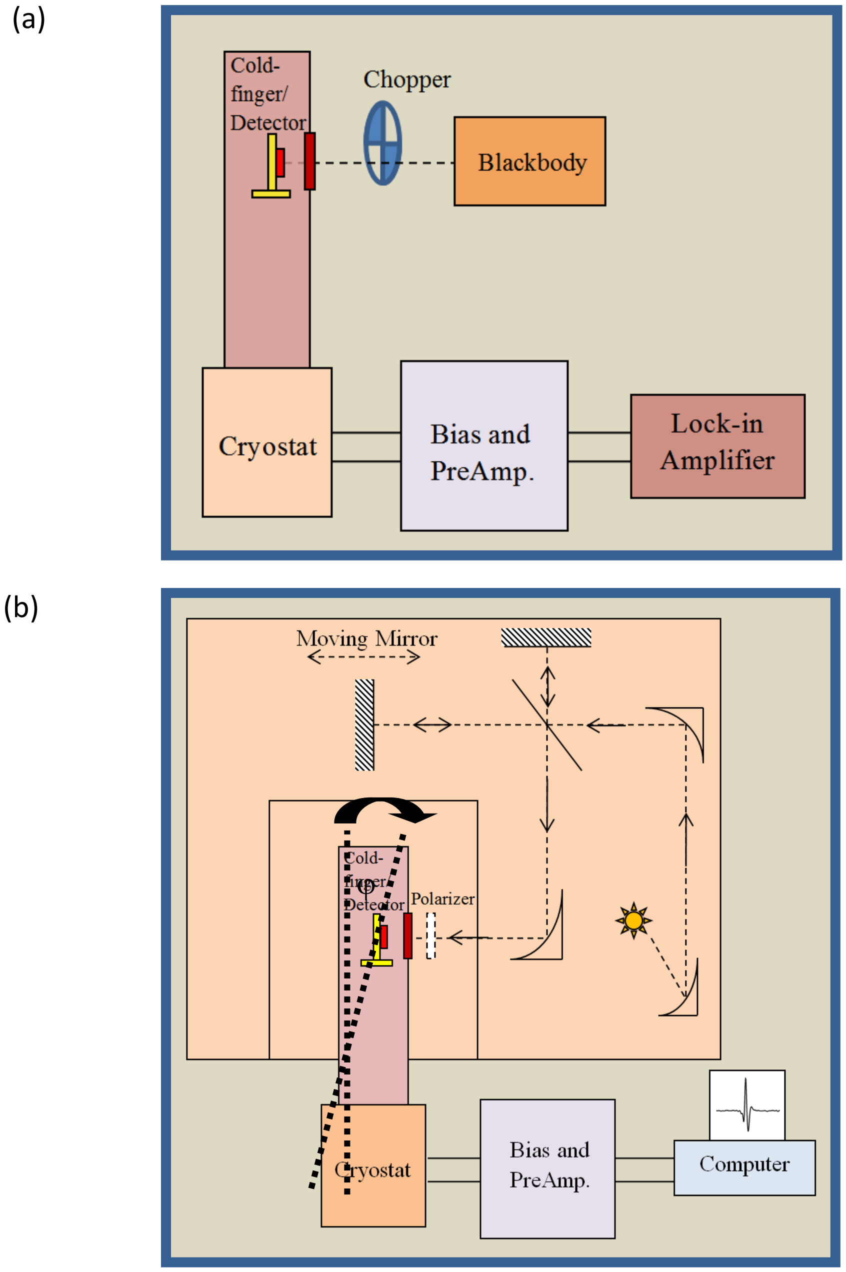

Figure S2 | Experimental setup (a) for measuring the blackbody responsivity, (b) for measuring the photocurrent spectrum at different incident angles.

3. Boundary conditions in the simulation of the PMC-QWIP pixel

In the PMC-QWIP device a single pixel consists of four complete patches, twelve incomplete patches, and vertically one dielectric spacer and one full reflection layer, as shown in Fig. 1 of the paper. Fig. S3 shows the cross-section of such a PMC-QWIP pixel in the simulation. Note that the fillers (green area in the figure) between the pixels are used to separate the adjacent pixels. Fig. S4 shows the comparison of $\overline{|E_z|^2}$ at the boundary conditions (BCs) between the perfect match layer (PML) and the periodic. It is found that when the BC is changed from the PML to the periodic, the $\overline{|E_z|^2}$ spectrum is almost the same, except for an occurrence of a weak peak at 12.3 μm and a redshift of about 0.1 μm for the peaks. Therefore, $\overline{|E_z|^2}$ in the QW layer is almost insensitive to the boundary conditions. It might be due to the fact that such a metal-patched MIM pixel is dominated by the cavity LSP mode and the periodicity-induced modes are only weakly coupled. Also, the distribution of the square of E_z in *XOY* plane at z=0.3 μm is simulated by using the periodic BC, as shown in Fig. S5, together with the contour of the MIM pixel structure. It is found that the square of E_z at the corners of the pixel is almost zero, the same as those with

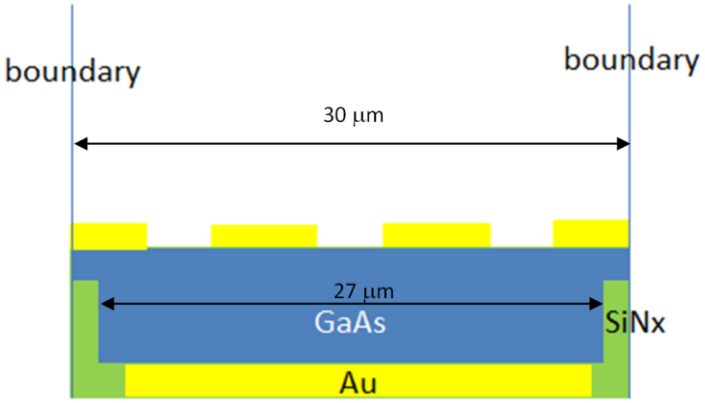

Figure S3| Cross-section of the PMC-QWIP pixel

the PML BC in the manuscript.

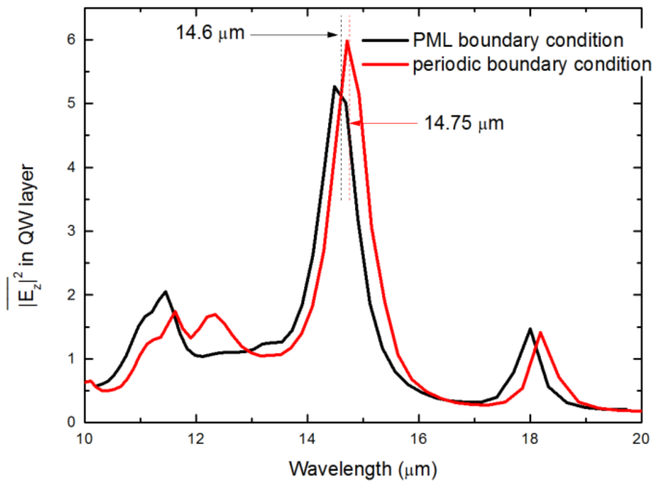

Figure S4| Comparison of $\overline{|E_z|^2}$ calculated with the PML and the periodic boundary conditions

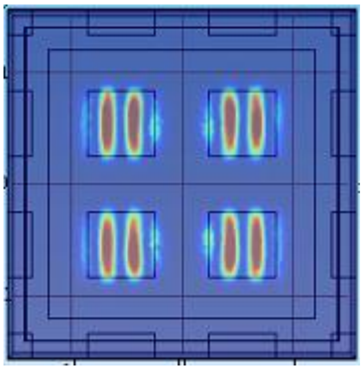

Figure S5| Distribution of the square of E_z in XOY plane at z=0.3 μm under the periodic BC

4. PL spectrum of the quantum well material

Fig. S6 describes the PL spectrum of the quantum well material; the substrate (GaAs) peak at 865 nm and the barrier layer peak ($Al_{0.15}Ga_{0.85}As$) at 762 nm are observed, as the result of the electronic transition from the conduction band to the valence band of GaAs and $Al_{0.15}Ga_{0.85}As$, respectively. Note that the thickness of $Al_{0.15}Ga_{0.85}As$ is so thin (100 nm) that the peak of $Al_{0.15}Ga_{0.85}As$ at 762 nm is weak, compared with the peak of GaAs at 865 nm.

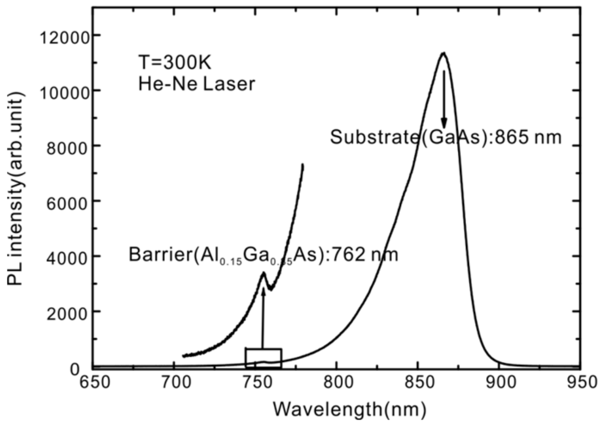

Figure S6| Room temperature photoluminescence of the quantum well material

5. Material model

Numerical simulation model is constructed with the finite element method, and the complex permittivity of the material is required. In this paper, we define the complex permittivity of Au and the quantum well according to the light absorption in the simulated wavelength range. We describe Au permittivity properties based on Drude mode:

$$\epsilon(\omega) = \epsilon_\infty - \frac{\omega_p^2}{\omega^2 + i\omega\gamma}; \quad \text{(S1)}$$

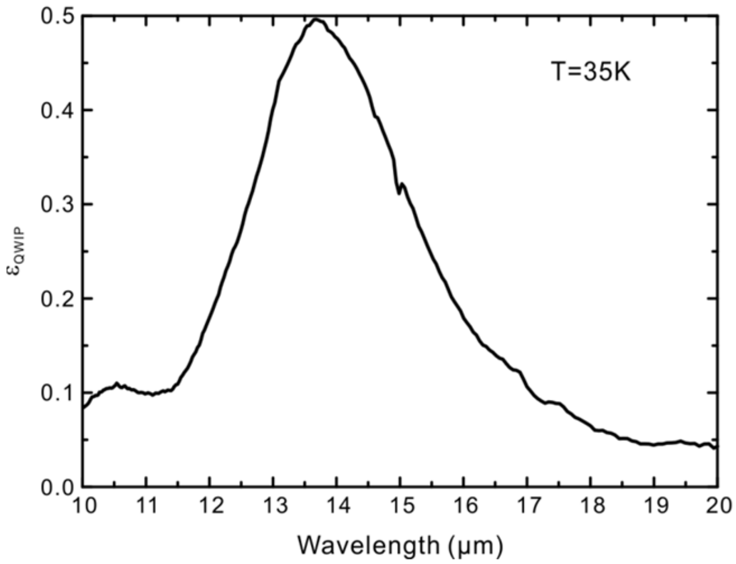

Figure S7| Imaginary part of the permittivity of the quantum well material in the *z*-direction, the data are taken from the experimental results at the working temperature of 35 K.

Here, ω_p = 1.63×10^{16} [rad/s] is the plasma frequency, γ = 0.74×10^{14} [rad/s] is the damping frequency, ε_∞ is the permittivity at high frequency and is taken as 1.

According to the quantum transition rule, the permittivity property of the quantum well is defined by the isotropic permittivity model[2]:

$$\varepsilon = \begin{bmatrix} \varepsilon_{\mathrm{GaAs}} & 0 & 0 \\ 0 & \varepsilon_{\mathrm{GaAs}} & 0 \\ 0 & 0 & \varepsilon_{\mathrm{GaAs}} + \varepsilon_{\mathrm{QWIP}} i \end{bmatrix} \quad \text{(S2)}$$

where $\varepsilon_{\mathrm{GaAs}}$ and $\varepsilon_{\mathrm{QWIP}}$ are the permittivity of intrinsic GaAs and the imaginary part of permittivity of the quantum well; their values are taken as 10.43 and from the experimental data shown in Fig. S7, respectively.

6. Semi-analytical method

We use a semi-analytical Fabry-Perot model to understand the resonance formation in the plasmonic microcavity. The resonance wavelength can be estimated via the F-P model. Because the impendence mismatch exists at the structure termination, the effective refractive indices at the two sides of the termination have abrupt change; photons in the microcavity will be reflected when they hit the termination. The resonance of the plasmonic microcavity occurs when the phase accumulation of light per round trip is a multiple of 2π, which can be expressed as follows[3, 4]:

$$k_0 n_{\mathrm{eff}} L + \phi_{\mathrm{r}} = m\pi \tag{S3}$$

where m=1, 2, 3… is the order of resonance, and ϕ_{r} is the phase of the reflection coefficient at the structure termination, n_{eff} is the effective refractive index of the MIM waveguide, L is the metal patch length along the propagation direction, and k_0 is the free space wave vector. According to k=2π/λ, the resonance wavelength can be defined as:

$$\lambda = \frac{2\pi n_{\mathrm{eff}} L}{m\pi - \phi_r}. \tag{S4}$$

The COMSOL platform is employed to numerically calculate the effective refractive index of the MIM waveguide mode and the modulus and phase of the reflection coefficient at the termination. First, we study the propagation characteristics of the waveguide mode, and the MIM waveguide structure is illustrated in Fig. S8a. A 0.89 μm-thick dielectric layer is sandwiched between the metal patch and the metal reflection layer. The dielectric layer consists of a 207 nm-thick quantum well active

layer, the top/bottom contact and the etch stop layer. The permittivity is defined by the experimental data in Fig. S7. The normal incident y-polarized light is coupled into the waveguide mode propagating along y-direction at the termination, and the simulated $\overline{|E_z|^2}$ at XOZ plane is shown in Fig. S8b at λ=15 μm. It is obvious to find that the optical energy is confined between the metal patch and the metal reflection layer, exhibiting the uniform distribution at the middle regions of the microcavity. By taking the metal patch length as 5, 6, 7, 8 μm, the effective refractive index of the waveguide and the lateral propagation length of the guided mode as the function of the wavelength can be calculated and are plotted in Figs. S8c and S8d. As one can see that the metal patch length has little influence on the index and the propagation length. In the wavelength range from 10 to 20 μm the effective refractive index is between 3.2 and 3.3, almost unchanged with the wavelength. However, a dip of the propagation length occurs at 13.5 μm, corresponding to the photon absorption of the quantum well (as shown in Fig. S7). Note that even at the absorption peak wavelength, the propagation length can reach more than 50 μm, indicating that the quantum well material behaves weak absorption characteristics. The optical mode decays very slowly in the waveguide, and photons can travel long enough distance.

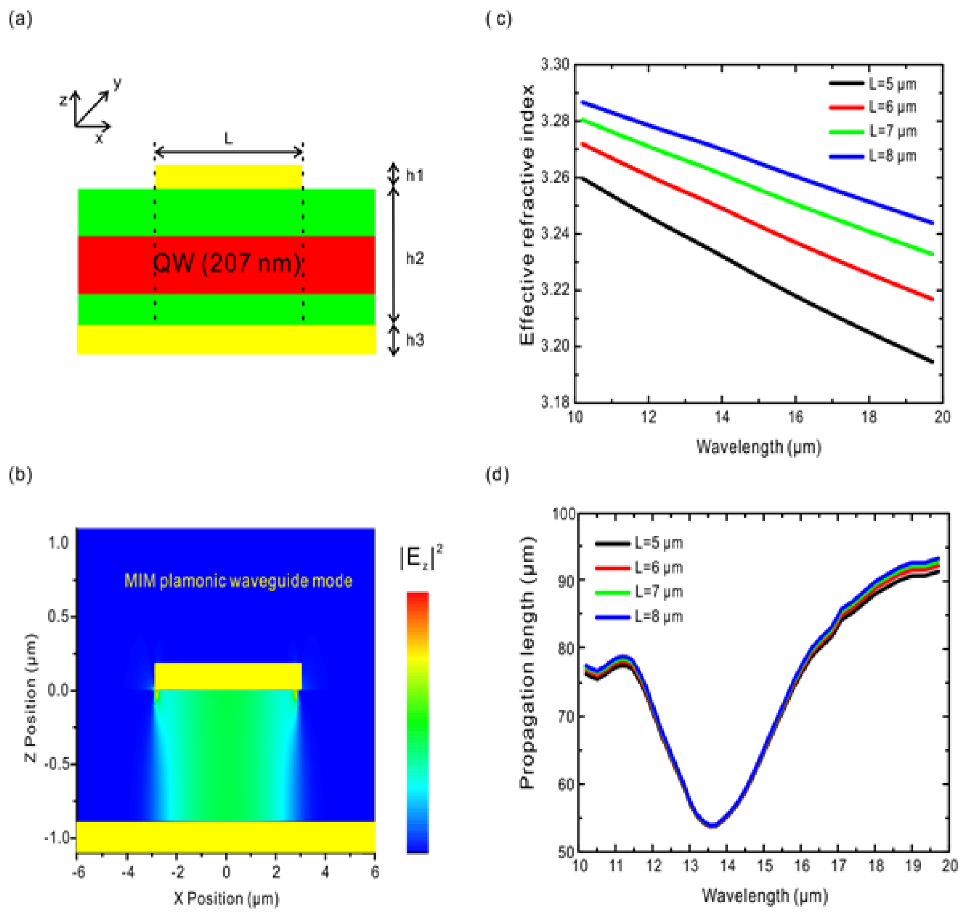

Figure S8| Characteristics of the MIM plasmonic waveguide mode. (a) Cross section of the MIM plasmonic waveguide consisting of a 0.89 μm-thick dielectric layer sandwiched by the metal patch with *L*=5.68 μm and *h*1=0.1 μm and the metal reflection layer with *h*3=0.1 μm. (b) Distribution of the square of electric component along the *z*-direction of the plasmonic MIM waveguide mode propagating along the *y*-axis at the wavelength of 15 μm. (c) Effective refractive index n_{eff} of the MIM waveguide mode with different metal patch lengths *L* as a function of the wavelength. (d) Propagation length L_m of the MIM waveguide mode with different metal patch length *L* as a function of the wavelength.

On the other hand, the reflection of the waveguide mode will occur at the termination due to the impendence mismatch. The schematic view of the *YOZ* cross

section and the simulated $\overline{|E_z|^2}$ are shown in Figs. S9a and S9b. The waveguide mode is excited at Port 1. Figs. S9c and S9d plot the modulus and phase of the reflection coefficient of the waveguide mode at the termination, and the metal patch lengths are taken as 5, 6, 7, 8 μm. The simulation shows that the modulus and phase of the reflection coefficient are insensitive to the metal length. However, as the wavelength gets longer, the modulus increases while the phase decreases. A larger reflectance implies that the photons' escape ratio from the microcavity to the free space becomes smaller, more number of the round reflections in the microcavity will be achieved; the amplitude of the standing wave will be stronger due to the constructive interference. In terms of photodetectors, that means the enhancement factor of the performance is higher, proves the experimental results that the responsivity enhancement factor of the resonance mode increases as the wavelength gets longer. In terms of the quality factor of the microcavity, the increased reflectance can prevent the cavity from radiation loss, leading to higher quality factor. It is noteworthy that both the effective index of the MIM waveguide and the phase of reflectance do not show the light absorption characteristics of the quantum well, which indicates that the insertion of the absorptive dielectric layer of the quantum well into the MIM structure does not lead to the shift of the resonance wavelength, in corresponding to the expression in Eq. (S4), that is, the resonance wavelength is determined by the metal patch length, the modulus and the phase of the reflection coefficient.

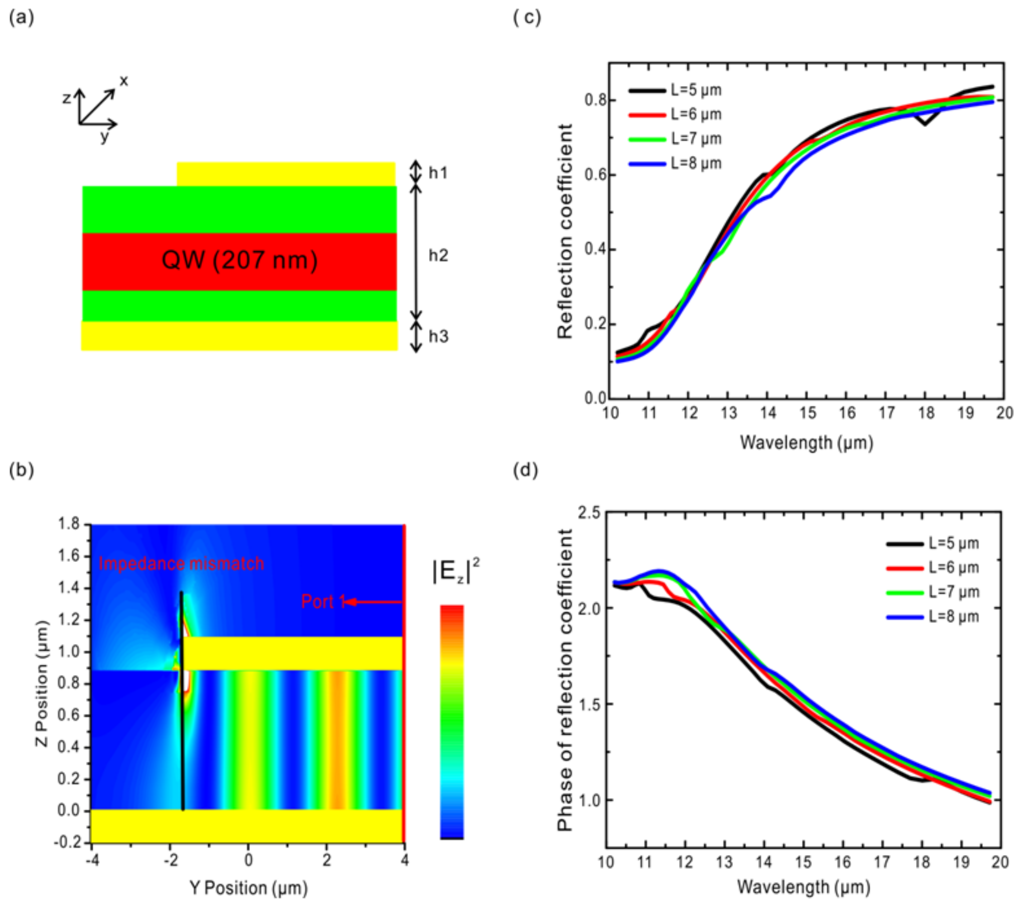

Figure S9| Characteristics of MIM plasmonic waveguide mode at the structure termination for the wavelength of 15 μm. (a) Schematic view of YOZ cross section. (b) Distribution of the square of electric component along *z* direction for the plasmonic waveguide mode being reflected at the termination as indicated by the black line, and waveguide mode is excited at Port 1. (c) The modulus of reflection coefficient of the MIM waveguide mode with different metal patch length *L* as a function of wavelength. (d) Phase of reflection coefficient of waveguide mode with different metal patch length *L* as a function of wavelength.

7. **Discussion about resonance on geometric parameters (metal patch length *L* and dielectric layer thickness *h*)**

As expected, the geometric parameters can be optimized to match a given wavelength. In other words, resonance on geometric parameters occurs when the maximum $\overline{|E_z|^2}$ in the QW layer is achieved, and $\overline{|E_z|^2}$ will fall quickly if the offset between the real and the ideal resonance parameters exists. To illustrate this offset effect of $\overline{|E_z|^2}$, the wavelength of 13 μm is chosen as the given wavelength where the experimental enhancement is the minimal and the offset is the maximum. Fig. S10a describes the map of $\overline{|E_z|^2}$ in the QW layer as a function of the dielectric layer thickness h and the metal patch length L at λ=13 μm. It is clear that the maximum

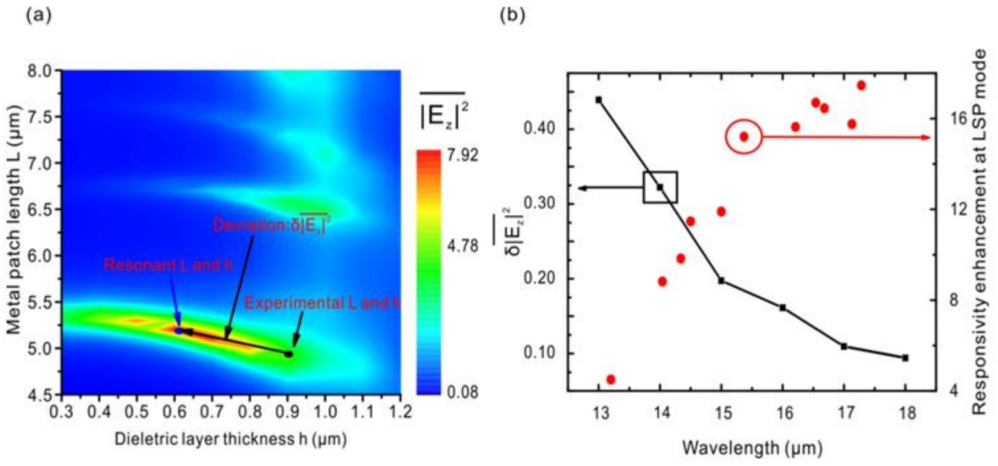

Figure S10| Effect of wavelength on the deviation $\delta\overline{|E_z|^2}$. (a) Map of $\overline{|E_z|^2}$ in the QW layer as a function of the dielectric layer thickness *h* and the metal patch length *s* at λ=13 μm, and the black and blue dots represent experimental and resonant points, respectively. (b) Deviation $\delta\overline{|E_z|^2}$ (black squares) and responsivity enhancement factor of the LSP mode (red dots) as a function of the wavelength

value of 7.9 of $\overline{|E_z|^2}$ is achieved when h and L are taken as 0.65 μm and 5.25 μm, and the resonance on s and h occurs. In other words, the 0.65 μm-thick dielectric layer should be chosen to realize the maximum enhancement of the performance. However, limited by the functional layers of the detector (top/bottom contact, active layer, etch stop layer), the thickness of the dielectric layer is difficult to reach the resonant point for the given wavelength. In fact, the experimental h is fixed at 0.887 μm instead of the corresponding resonant thickness, which indicates that an offset will exist, which can be defined as $\delta\overline{|E_z|^2}$:

$$\delta\overline{|E_z|^2}(\lambda) = 1 - \frac{\overline{|E_z|^2}_r}{\overline{|E_z|^2}_i} \tag{S5}$$

where $\overline{|E_z|^2}$ is the average of the square of electric component along the z-direction in the QW layer, and subscripts r and i represent the real and the ideal geometric parameter, respectively. To explain the experimental relationship between the responsivity enhancement factor of the LSP mode and the wavelength, we plot $\delta\overline{|E_z|^2}$ as a function of the wavelength, as shown in Fig. S10b. The results show that as the wavelength gets longer $\delta\overline{|E_z|^2}$ tends to be smaller, approaching zero. This means that as the offset resulting from the mismatch between the real and the ideal resonant parameters becomes smaller, the experimental parameter is close to the ideal one, in good agreement with the relationship between the responsivity enhancement factor of the LSP mode and the wavelength, as shown in Fig. S10b as red dots. Thus it demonstrates that the increased responsivity enhancement factor at longer wavelength LSP mode in experiment is resulted from the closer of the geometrical parameters to the resonant conditions.

Movie legend:

Supplementary Movie 1 | A live process in which photons are captured into the plasmonic microcavities from free space and forming strong LSP mode.

Supplementary Movie 2 | A comparison of imaging between the PMC-QWIP and the Std-QWIP with a moving cross hole screen placed in front of a blackbody light source. The imaging quality has been greatly improved with the PMC-QWIP.

References

1. Levine BF. Quantum-Well Infrared Photodetectors. *J Appl Phys* **74**, R1-R81 (1993).

2. Zhao FY, Zhang C, Chang HT, Hu XH. Design of Plasmonic Perfect Absorbers for Quantum-well Infrared Photodetection. *Plasmonics* **9**, 1397-1400 (2014).

3. Dorfmuller J*, et al.* Fabry-Perot Resonances in One-Dimensional Plasmonic Nanostructures. *Nano Lett* **9**, 2372-2377 (2009).

4. Bin Hasan S*, et al.* Relating localized nanoparticle resonances to an associated antenna problem. *Phys Rev B* **84**, (2011).
